# A retrospective cohort study investigating the association of environment, milk performance and udder health with the yield and solid content of first-milking colostrum in Holstein dairy cows

**DOI:** 10.1186/s12917-025-04909-3

**Published:** 2025-07-11

**Authors:** Gonçalo Pereira, Ricardo Bexiga

**Affiliations:** 1 CIISA– Centre for Interdisciplinary Research in Animal Health, Faculty of Veterinary Medicine, University of Lisbon, Lisbon, Portugal; 2 Associate Laboratory for Animal and Veterinary Sciences (AL4AnimalS), Lisbon, Portugal

## Abstract

**Background:**

The administration of high-quality colostrum is widely recognized as crucial for the health and survival of neonatal calves, yet identifying factors influencing colostrum yield and solid content has remained a challenge. With dairy farmers facing increasing difficulties in maintaining a year-round supply of high-quality colostrum, this retrospective cohort study evaluated the association between environmental, milk performance, and udder health factors with colostrum yield and solid content (as measured by Brix % determination) in 1731 lactations of Holstein dairy cows (583 primiparous and 1148 multiparous) on a commercial farm.

**Results:**

In primiparous cows, colostrum yield was associated with the time interval between calving and colostrum collection (as estimated by whether colostrum was collected during morning or afternoon milking), while lower colostrum solid content was associated with higher occupancy of the close-up pen. In multiparous dairy cows, both colostrum yield and solid content were associated with lactation number (higher lactations associated with lower yield but with higher solid content), dry period length (longer dry periods associated with higher yield and higher solid content), and whether colostrum was collected during morning or afternoon milking. A lower colostrum solid content of multiparous cows was also associated with higher occupancy of the close-up pen and a higher colostrum yield was associated with higher THI during the 21 days before calving. Moreover, greater milk yield at dry-off was associated with lower colostrum yield and a longer previous calving interval was associated with greater colostrum yield. These findings highlight the benefits of longer dry periods and calving intervals for colostrum production, the need to revise dry-off practices for high-yield dairy cows, and the importance of addressing overpopulation in close-up pens, especially in heat-stressed herds with higher variation in close-up pen occupancy. This work also reinforces the importance of shorter intervals between calving and colostrum collection for higher solid content of colostrum in multiparous cows.

**Conclusions:**

Overall, this study enhances understanding of the factors influencing colostrum production and provides valuable insights for developing improved colostrum management strategies.

## Background

The syndesmochorial placenta in cattle prevents the *in utero* transmission of protective immunoglobulins (Ig) from maternal blood to the fetus, which results in calves being born without circulating Ig and relying entirely on the intestinal absorption of maternal Ig from colostrum for protection against infectious diseases, as reviewed by [1]. This is evidenced by the decreased preweaning disease probability and mortality, as well as increased average daily gain in calves with excellent transfer of passive immunity (TPI) [2]. Therefore, proper administration of high-quality colostrum is widely recognized as the main factor determining the health and survival of neonatal calves [3].

To achieve proper TPI, a colostrum management program should consider the timing and the amount of Ig administered. The two main factors affecting the amount of Ig provided are the quantity and quality of colostrum provided. In terms of timing and quantity, the colostrum should be given at 10% of the calf’s body weight (about 4 L for Holstein calves, approximately 40 kg bodyweight) within the first two hours of life, followed by a second similar-sized feeding within 12 h old, as reviewed by [3].

Colostrogenesis refers to the prepartum process by which immunoglobulins are transferred from the maternal bloodstream into mammary secretions. This transfer occurs via transcytosis and typically begins approximately 4 to 5 weeks before calving, concluding relatively abruptly at parturition [4].

Immunoglobulins are especially abundant, comprising 70–80% of the total protein content, with IgG1 being the predominant isotype [5]. In addition to immunoglobulins, the mammary gland accumulates various bioactive components and somatic cells, mainly leukocytes such as macrophages and neutrophils, which contribute to the newborn calf’s immune protection. Moreover, colostrum differs markedly from mature milk in composition as it contains higher levels of protein, sodium, and chloride, and lower concentrations of potassium and lactose [5].

Although colostrum contains many important immunological and nutritional components, its quality is traditionally assessed based on IgG concentration, with high-quality colostrum defined as having more than 50 g/L of IgG [6]. On commercial dairy farms, colostrum quality has been assessed using a Brix refractometer, which measures the percentage of solids in a solution to indirectly estimate the IgG level in colostrum. Currently, a Brix value above 22% is considered indicative of high-quality colostrum (IgG > 50 g/L), as the industry standard [7]. Colostrum quality can also vary significantly between cows and although much research has been conducted on factors associated with colostrum quality in cows, little is understood regarding what conjunction of factors is causing the variability seen in colostrum quality [8]. Among the most studied factors are parity [9], duration of the dry period [10], overall performance in the previous lactation [11], prepartum nutrient intake [12] and udder health during the previous lactation, as reviewed by [13]. Moreover, it is also suggested that the quantity and quality of colostrum are affected by environmental factors such as temperature-humidity index (THI) [14], and photoperiod during the dry period [15].

In addition to the importance of administering good quality colostrum to calves within the first 24 h of life, recent studies have shown that extending colostrum feeding or feeding transition milk after gut closure and during the first two weeks of life still offers benefits. Adopting this type of feeding during the early days of a calf’s life proved beneficial to calf health (fewer days with a rectal temperature ≥ 39.4⁰C and lower prevalence of diarrhea and pneumonia) as well as to postweaning performance (average daily gain) [16]. However, it will also lead to a need for increased storage capacity of good quality colostrum as consumption will also be greater.

Given the individual and seasonal variability in colostrum yield and composition, maintaining an adequate supply of high-quality colostrum year-round remains a challenge for commercial dairy producers [14]. This issue can be addressed through meticulous planning and the creation of a colostrum bank to store colostrum or through the use of colostrum replacements when necessary. However, the cost-benefit of using colostrum replacements is questionable due to cost and highly variable performance among different products [17].

Given the increased challenge for dairy farmers to maintain an adequate supply of high-quality colostrum year-round, this study aims to evaluate the associations between environmental, milk performance and udder health factors with the quantity and quality of colostrum produced by Holstein dairy cows on a commercial dairy farm, with the goal of identifying potential modifiable factors that can inform effective strategies to improve colostrum supply.

## Results

### Study population

The study population was composed by cows in the first (*n* = 583), second (*n* = 467), third (*n* = 336), fourth (*n* = 211), fifth (*n* = 89), and sixth or higher (*n* = 45) lactations. The average THI cows experienced during the last 21 days before calving was 73.04 ± 0.14, with 52.2% (904/1731) of the observations belonging to a cow exposed to an average THI greater than or equal to 72 in the 21 days before calving. The average occupancy in the close-up pen during the 21 days prior to the calving date was 58.83 ± 0.51 cows. This corresponds to an average of 25.63 m² of resting space and 0.94 m of feeding space per cow. However, despite these averages being within industry targets commonly used by AHDB [18] (0.85 m of feed space and 15 m² of lying space per cow), and although all cows had more than 15 m² of lying space, 43.5% of the cows (754 out of 1,732) experienced insufficient feeding space (< 0.85 m) during the 21 days before calving. The average calving age for primiparous cows was 24.17 ± 0.08 months with a minimum of 21.3 and a maximum of 35.8 months. The average daily milk yield before dry-off was 24.38 ± 0.27 kg. The average dry period length was 50.57 ± 0.57 days with a minimum of 10 and a maximum of 201 days. Also, the average previous calving interval was 407.28 ± 1.94 days. Regarding udder health, on average, each observation had a previous lactation with 2.31 ± 0.09 monthly DHI tests with somatic cell count (SCC) linear score (LS) ≥ 4 and the average LS of SCC in the last DHI test before dry-off (LLS) was 3.19 ± 0.05.

### Colostrum production

The average first milking colostrum yield for all animals in the study was 4.98 ± 0.09 kg, a complete lack of colostrum (0 kg) was recorded for 68 animals and the average %Brix was 24.31 ± 0.10%. Primiparous cows showed the highest mean colostrum yield (5.52 ± 0.18) while sixth or higher lactation cows showed the lowest (4.42 ± 0.66). Regarding %Brix, older cows (sixth or greater lactation) exhibited the highest %Brix (26.56 ± 0.73) and second lactation cows showed the lowest (22.93 ± 0.23). Moreover, 62.6% (1084/1731) (56.4% in primiparous and 65.7% in multiparous) of the cows had a colostrum yield < 6 kg, which is the amount of colostrum generally accepted as needed for two colostrum feedings. Additionally, of the 647 cows yielding 6 or more kg of colostrum, only 480 had a %Brix equal or greater to 22%, meaning that only 27.7% (480/1731) had both sufficient quantity and quality compatible with high-quality colostrum.

### Colostrum yield in primiparous

Colostrum yield in primiparous cows was only associated with whether colostrum was collected in the morning or afternoon milking (*P* = 0.009) (Table 1) with primiparous cows having their colostrum collected in the afternoon milking showing higher mean yield than those whose colostrum was collected in the morning milking. Also, noteworthy that the average daily maximum THI for the 21 days before the calving date (THI21) was not associated with colostrum yield in primiparous.

**Table 1:** Effects retained in the final multivariable model for variables associated with colostrum yield (kg)

	Primiparous		Multiparous		
	LSM (95% CI)	*P*	Estimate (95% CI)	LSM (95% CI)	*P*
Milking collection^1^		0.009			0.022
Morning	5.26 (4.90 to 5.62)^a^			4.69 (4.26 to 5.12)^a^	
Afternoon	6.17 (5.60 to 6.75)^b^			4.11 (3.64 to 4.58)^b^	
THI21 (unit)			0.070 (0.025 to 0.116)		0.002
DPL (linear) (d)	-		0.174 (0.117 to 0.230)		< 0.001
DPL (quadratic) (d)	-		-0.0009 (-0.0012 to -0.0005)		< 0.001
MYDRY (kg)	-		-0.077 (-0.115 to -0.039)		< 0.001
PCI (d)	-		0.008 (0.003 to 0.012)		< 0.001
LN	-				0.032
2				5.14 (4.79 to 5.63)^a^	
3				4.73 (4.26 to 5.19)^a^	
4				4.47 (3.87 to 5.06)^ab^	
5				3.73 (2.88 to 4.58)^b^	
6+				3.89 (2.59 to 5.18)^ab^	

Within a column, least squares means without a common superscript differ (*P* < 0.05) among the category level

Abbreviations: THI21 = Average daily maximum temperature-humidity index for the 21 days before the calving date; DPL = Dry period length; MYDRY = average daily milk yield in kg in the 4 days before dry-off; PCI = Previous calving interval in days; LN = Lactation number; CI = Confidence interval; LSM = Least Squares Means

^1^ Whether colostrum was collected in the morning or afternoon milking

### Colostrum yield in multiparous

Colostrum yield in multiparous cows was associated with THI21 (*P* = 0.002), dry period length (*P* < 0.001), daily milk yield before dry-off (*P* < 0.001), previous calving interval (*P* < 0.001), whether colostrum was collected in the morning or afternoon milking (*P* = 0.022), and lactation number (*P* = 0.032) (Table 1). For each unit increase in THI21, multiparous cows yielded 0.071 ± 0.023 kg more colostrum. Regarding the quadratic effect of dry period length, maximal colostrum yield was predicted at a dry period length of 100 days. For each kg increase in daily milk yield before dry-off, multiparous cows yielded 0.077 ± 0.019 kg less of colostrum. For each day increase in the previous calving interval, multiparous cows yielded 0.008 ± 0.002 kg more of colostrum. Additionally, multiparous cows having their colostrum collected in the morning had a higher yield (4.69 ± 0.22 kg) than multiparous whose colostrum was collected in the afternoon milking (4.11 ± 0.24 kg). Regarding lactation number, second and third lactation cows exhibited higher mean colostrum yield than fifth lactation cows whereas fourth and sixth or greater lactation cows did not differ from any other lactations.

### Colostrum solid content in primiparous

Colostrum solid content in primiparous cows was only associated with average occupancy of the close-up pen for the 21 days before the calving date (*P* = 0.045) (Table 2), for each cow increase in the close-up pen, primiparous cows yielded colostrum with 0.020 ± 0.001 less %Brix. Also, noteworthy that neither THI21 nor age at calving were associated with colostrum solid content in primiparous.

**Table 2:** Effects retained in the final multivariable model for variables associated with colostrum solid content (%Brix)

	Primiparous		Multiparous		
	Estimate (95% CI)	*P*	Estimate (95% CI)	LSM (95% CI)	*P*
OCC21	-0.020 (-0.039 to -0.001)	0.045	-0.015 (-0.028 to -0.002)		0.024
Milking collection^1^					0.002
Morning				24.44 (23.97 to 24.90)^a^	
Afternoon				25.33 (24.82 to 25.84)^b^	
DPL (d)	-		0.0155 (0.0009 to 0.0301)		0.038
LN	-				< 0.001
2				23.06 (22.62 to 23.50)^a^	
3				24.09 (23.57 to 24.60)^b^	
4				25.17 (24.51 to 25.82)^c^	
5				25.46 (24.52 to 26.40)^c^	
6+				26.64 (25.25 to 28.03)^c^	

Within a column, least squares means without a common superscript differ (*P* < 0.05) among the category level

Abbreviations: OCC21 = Average occupancy (number of cows) of the close-up pen for the 21 days before the calving date; DPL = Dry period length; LN = Lactation number; CI = Confidence interval; LSM = Least Squares Means

^1^ Whether colostrum was collected in the morning or afternoon milking

### Colostrum solid content in multiparous

Colostrum solid content in multiparous cows was associated with average occupancy of the close-up pen for the 21 days before the calving date (*P* = 0.024), lactation number (*P* < 0.001), whether colostrum was collected in the morning or afternoon milking (*P* = 0.002), and dry period length (*P* = 0.038) (Table 2). Regarding close-up pen occupancy, for each cow increase, multiparous cows yielded colostrum with 0.015 ± 0.006 less %Brix. Regarding lactation number, second lactation cows yielded colostrum with lower solid content (23.06 ± 0.225%Brix) than any other lactations, and third lactation cows (24.09 ± 0.263%Brix) yielded colostrum with lower solid content than cows in their fourth (25.17 ± 0.331%Brix), fifth (25.46 ± 0.479%Brix) and sixth or over (26.64 ± 0.709%Brix) lactation. Additionally, multiparous cows having their colostrum collected in the morning had lower solid content (24.44 ± 0.236%Brix) than multiparous whose colostrum was collected in the afternoon milking (25.33 ± 0.259%Brix). Finally, for each day increase in dry period length, multiparous cows yield colostrum with 0.0155 ± 0.0074 more %Brix. Also, noteworthy that neither THI21, average daily milk yield during the previous calving interval, number of DHI > 4LS or the LLS were associated with colostrum solid content in multiparous cows.

## Discussion

High quality colostrum is essential for the successful transfer of passive immunity, which is critical for the health and survival of newborn calves and the overall profitability of dairy farms [2, 3, 19]. This study investigates the environmental, milk performance, and udder health factors affecting the yield and solid content of first-milking colostrum in Holstein dairy cows, aiming to identify modifiable factors which could potentialy inform strategies to improve colostrum availability.

Identifying these factors is particularly important, as in the present study, only 27.7% of cows produced colostrum with both the yield and solid content required for high-quality standards (≥ 6 kg and ≥ 22% Brix) [20, 21]. Additionally, when considering only yield, 62.6% of cows produced less than 6 kg of colostrum, aligning with [14]. However, those authors observed that inadequate colostrum yield was more prevalent in primiparous cows compared to multiparous cows (73.4% in primiparous vs. 61.5% in multiparous). In contrast, the present study found a higher proportion of multiparous cows with insufficient colostrum volumes (56.4% in primiparous vs. 65.7% in multiparous). These differences might suggest the influence of farm-specific factors on colostrum yield, which require further investigation.

In primiparous dairy cows, the present study showed that colostrum yield was associated with whether colostrum was collected during morning or afternoon milking, while colostrum solid content was linked to the average occupancy of the close-up pen during the 21 days before calving. In multiparous dairy cows, both colostrum yield and solid content were associated with lactation number, dry period length, and the timing of colostrum collection (morning vs. afternoon). Additionally, like primiparous cows, colostrum solid content in multiparous cows was associated with the average occupancy of the close-up pen during the 21 days before calving. Lastly, in multiparous cows, colostrum yield was also associated with the average THI21, the average daily milk yield during the 4 days before dry-off, and the length of the previous calving interval.

In the present study, there was a clear association between colostrum yield and solid content in multiparous cows and respective lactation number. Cows in their second and third lactation cows yielded more colostrum than fifth lactation cows, while exhibiting lower colostrum solid content compared to cows in their fourth or greater lactations. This result aligns with [14], who also found that second lactation cows yielded the most colostrum while having the lowest solid content, whereas fifth or greater lactation cows yielded the least colostrum while having the highest solid content. Moreover, despite also observing the positive association between lactation number and colostrum solid content [22], did not observe any association between lactation number and colostrum yield among multiparous cows. The positive association between lactation number and colostrum solid content was also observed by [23], as in their work cows in fifth or greater lactation had greater colostrum IgG concentration than lower-parity animals.

Colostrum yield in multiparous cows was also positively correlated with the average THI21. This aligns with [14], who observed that colostrum yield in multiparous Holstein cows increased as THI rose, and [15], who reported a similar positive correlation between colostrum yield in Jersey cows and maximum weekly THI. This positive relationship between THI and colostrum yield is challenging to explain, as high THI typically reduces dry matter intake [24], and prepartum nutrient intake is known to influence colostrum production in ruminants [12]. Additionally, research indicates that cooling heat-stressed dry cows increases both colostrum yield and IgG concentration [25]. These authors also observed that the lower colostrum yield and IgG concentration in heat-stressed cows were only partially attributable to reduced feed intake, as pair-fed cooled cows had colostrum yields and IgG concentrations comparable to those of ad libitum-fed cooled cows.

One possible explanation for the seemingly counterintuitive positive association between THI21 and colostrum yield is the fact that, in many cases, as THI increases, the photoperiod also increases. Longer photoperiods have been linked to higher colostrum yield considering that colostrum yield is typically lowest in the winter. Supporting this, a year-long study on a Texas dairy herd described that colostrum yield in multiparous cows was strongly correlated with photoperiod both one month before and at the time of calving [15]. Similarly [14], reported increased colostrum yield in multiparous Holstein cows with higher light intensity 14 days before calving.

Although the initial objective was to evaluate the association between photoperiod and colostrum yield, photoperiod and THI were highly correlated in the present dataset, leading to the retention of only THI in the final models to avoid multicollinearity issues. This underscores the need for controlled studies that can separate the effects of photoperiod and THI to determine if a causal relationship exists [13].

The association between colostrum collection timing (morning vs. afternoon) and both yield and solid content is likely due to the longer interval between calving and colostrum collection in cows whose colostrum was collected in the morning compared to those collected in the afternoon. This association varied between primiparous and multiparous cows. In primiparous cows, morning collection resulted in a lower yield but a similar solid content compared to afternoon collection. In multiparous cows, however, morning collection was associated with a higher yield but a lower solid content than afternoon collection.

For multiparous cows, the higher yield and lower solid content in morning collections may be explained by a dilution effect from the mixing of colostrum with transition milk when the interval between calving and collection exceeds 8 h [13]. This dilution effect occurs due to the progression from colostrum production (lactogenesis I) to copious milk production (lactogenesis II), which typically begins around the time of parturition [26]. The dilution effect of time to colostrum collection was also observed by [23] with a time to collection greater than 12 h resulting in a colostrum with lower solid content. Although 8 and 12 h intervals between calving and colostrum collection are very long, in the present study, a cow that calved from 6:00 p.m. would have had her colostrum collected at 10:30 a.m. the following day. Therefore, the dilution effect associated with extended time to collection remains a relevant consideration when interpreting colostrum yield and composition.

In primiparous cows, the lack of solid content differences between morning and afternoon collections may suggest a delayed onset of lactogenesis II, aligning with findings from a review that reported a higher incidence of delayed lactation onset among primiparous women compared to multiparous women in multiple prospective studies [27]. Moreover, the possibility of nighttime calf nursing prior to collection may explain the lower colostrum yield observed during morning collections in primiparous cows. Despite a similar or even stronger association might be expected in multiparous cows, this effect may be mitigated by their faster transition from colostrum production to copious milk production, which could replace any colostrum nursed with transition milk before collection. However, whether colostrum was collected in the morning or afternoon serves as an imperfect proxy for the calving-to-collection time interval. Therefore, these results should be confirmed in a setting where the actual elapsed time is accurately accounted for.

The colostrum solid content of both primiparous and multiparous cows was lower when the average occupancy of the close-up pen during the 21 days prior to calving was greater. This finding contrasts with [28], who reported that reducing the stocking density of the close-up pen (120%, 100%, and 80% headlock and stall occupancy) did not affect colostrum yield or Brix percentage. Although finding no effect of stocking density on colostrum solid content, these authors observed that lower stocking densities could increase lying and ruminating behaviours in prepartum dairy cows [28]. Additionally, their study included only 48 cows, which, given the many factors influencing colostrum solid content, may have limited its ability to detect significant differences.

Overall, average close-up pen occupancy in the 21 days before calving may be a key factor influencing colostrum solid content, particularly in herds affected by heat stress, as in the current study. In the Northern Hemisphere, dairy herds calving year-round experience heat-stress-related declines in conception rates from June to October, resulting in higher calving rates in autumn [29]. Facilities are typically designed for average occupancy across the year, so close-up pen occupancy in autumn may be nearly double that of spring and summer. This potential overcrowding can impact colostrum solid content due to limited bedding area and feeding space, highlighting the need for further investigation.

In the present study, a quadratic relationship was identified between dry period length and colostrum yield, with the maximum yield predicted at 100 days. This finding aligns with [30], who described the negative impact of both excessively short and overly long dry periods on subsequent lactation milk yield. Moreover, this association between dry period length and colostrum yield agrees with observational data [14], highlighting that colostrum yield increased as dry period length increased, categorized as < 47, 47–67, or > 67 days. Similarly, another research team observed that a shorter dry period (28 days) resulted in decreased colostrum volume compared to a 50-day dry period [31]. Given that most mammary cell proliferation occurs during the dry period [32], it was suggested that reduced dry period length could affect cell proliferation or function during colostrogenesis [14].

Regarding colostrum solid content, the observed positive association with dry period length was also reported by [14], who found that colostrum Brix % was higher for cows with a dry period > 67 days compared to those with < 47 or 47–67 days. However [33], reported that while omitting the dry period entirely was associated with lower IgG and IgM concentrations in colostrum, a 30-day dry period did not result in lower IgG or IgM concentrations compared to a 60-day dry period. Overall, these findings suggest that dry periods longer than 60 days may need to be considered to observe a consistent positive association with colostrum solid content. However, the reliability of the predicted relationships between dry period length and colostrum yield and solid content should be interpreted with caution, as the present study was not specifically designed to assess this variable. Additionally, the distribution of observations across dry period categories was unbalanced, with data from 666, 343, and 139 lactations corresponding to < 47, 47–67, and > 67 days, respectively, based on the classification used by [14].

Regarding the negative association identified between milk yield at dry-off and colostrum yield in multiparous cows, higher milk yields at dry-off are associated with delayed mammary involution [34], increased stress markers [35], and more severe metabolic and inflammatory changes around dry-off compared with low-producing cows [36]. Additionally, cows with high milk yield at dry-off also exhibited reduced lying times, which may indicate discomfort due to the accumulating milk in the udder [37]. The negative association observed in the present work between milk yield at dry-off and colostrum yield in multiparous cows can be the result of either delayed mammary involution, increased stress or inflammatory markers in colostrogenesis and should be further explored.

A positive association between the previous calving interval and colostrum yield was also observed, with earlier research identifying a similar relationship between longer calving intervals in primiparous cows and higher milk yields in the subsequent lactation [38]. This association has been attributed to the additional time provided by longer calving intervals, which allows cows to recover body reserves following the previous calving [39]. Similarly, we propose that, within the range of previous calving intervals studied, extended intervals may promote a better metabolic state during colostrogenesis, thereby increasing colostrum yield in the subsequent lactation.

Despite the well-reviewed association between udder health and milk yield [40], as well as the reduced colostrum yield observed in persistently infected mammary glands compared to uninfected glands [41], no associations were found between the number of DHI > 4LS, the LLS, and colostrum yield or solid content.

Although counterintuitive, these results align with the findings of [42], who reported that quarter-infection status in Holsteins did not significantly influence antibody concentrations in colostrum. Similarly [43], found no association between clinical mastitis in the previous lactation and colostrum yield, Brix percentage, or IgG concentrations. However, in the present study, most cows would have received antibiotic dry cow therapy and as [44] highlighted, this type of management minimizes the effect of subclinical mastitis at dry off and further investigations should be undertaken when exploring the associations of udder health at dry off with colostrum yield and solid content while considering selective dry cow therapy.

While there appears to be a lack of association between udder health and colostrum yield or solid content, maintaining udder health during the dry period should remain a priority. Not only may udder health affect colostrum components in ways not captured by standard solid content measures, but also its importance extends beyond considerations for colostrum production alone.

## Conclusion

In conclusion, although relying on time of colostrum collection (morning vs. afternoon) is an imperfect proxy for the calving-to-collection interval, this study supports the association between the calving-to-conception interval and colostrum yield in both primiparous and multiparous cows. Additionally, occupancy of the close-up pen was found to be relevant to colostrum solid content in both cow groups. These findings highlight the need to address overpopulation in close-up pens, namely in herds exposed to heat stress where higher occupancy variation will occur throughout the year while also reinforcing the importance of shorter intervals between calving and colostrum collection. Colostrum yield of multiparous cows was also associated with lactation number, average THI21, dry period length, milk yield at dry-off, and the length of the previous calving interval. These findings emphasize the role of extended dry periods and calving intervals in supporting optimal colostrum production, as well as the need to revisit dry-off practices of high yielding dairy cows. Overall, this study enhances understanding of the factors influencing colostrum production and provides valuable insights for developing improved colostrum management strategies.

## Methods

### Study design

This retrospective cohort study used environmental, milk performance, and udder health data from a commercial dairy farm between April 2021 and June 2024. Therefore, this analysis did not require approval by an Institutional Animal Care and Use Committee or Institutional Review Board.

### Farm and animals

Data were retrieved from a 800 milking Holstein-Friesian cow commercial dairy herd located in Portugal. The farm was selected for the present study because its overall management of dry cows remained consistent throughout the study period, and it is located in a region particularly affected by heat stress, with maximum daily THI values exceeding 72 from July to October. Cows on this farm were milked three times daily with an average 305-d milk yield of 11,853 kg in 2023. The farm used DHI services including the individual-cow SCC for each monthly DHI sampling of every milking cow. The data set included records from 1731 lactations, 583 from primiparous and 1148 from multiparous cows with 539 cows being included in more than one lactation.

### Dry-off, Close-up and after calving management

Although calving occurs year-round, this farm exhibits a marked increase in calvings during the autumn. This pattern is attributed to the effects of heat stress, which delays conception during the summer months due to elevated temperatures. As a result, more cows become pregnant during the cooler months of November, December, and January. This seasonal variation in conception leads to significant fluctuations in the occupancy of the close-up pen throughout the year. Cows are abruptly dried off weekly at approximately 45 days before their expected calving day and moved to a far-off dry cow field. Approximately 21 days before expected calving date, dry cows and pregnant heifers are vaccinated against E. coli adhesins, rotavirus and coronavirus, to raise respective antibodies in their colostrum and moved to a naturally ventilated straw-bedded pack barn and offered a corn silage and chopped straw–based TMR (Table 3), with anionic salts (DCAD: −10.00 mEq/100 g of DM), once a day. The close-up barn has 1508 m2 of resting and 55.5 m of feeding space.

Regarding after calving care, newborn calves born during the day (7:00 a.m. to 7:00 p.m.) were removed from the maternity area as soon as possible after birth while calves born during the evening or night were removed at 7:00 a.m. the following morning.

**Table 3:** Average nutrient concentrations of the total mixed ration delivered to the close-up pen

Nutrient concentrations^1^ (% of DM)	
% DM	49.37 ± 1.87
Crude protein	14.08 ± 0.17
Crude fat	3.08 ± 0.09
Starch	21.06 ± 1.26
Neutral detergent fiber (NDF)	44.85 ± 1.70
Acid detergent fiber (ADF)	27.79 ± 1.10
Acid detergent lignin (ADL)	5.36 ± 0.24
Ash	6.71 ± 0.17

^1^Analyzed by Near-infrared spectroscopy

DM = Dry matter

### Environmental data

Temperature and relative humidity were obtained at 15-minute intervals from a meteorological station inside one of the dairy’s milking cow barns. The THI was calculated according to [45]:

THI = (1.8 × T + 32)– [(0.55–0.0055 × RH) × (1.8 × T– 26)],

where T is the air temperature (°C) and RH is the relative humidity percentage. The maximum THI was recorded for each day.

The initial plan was to include not only THI data but also daily minutes of sunlight as well as solar radiation, but these two variables were highly correlated with THI (Pearson correlation coefficients > 0.75) and could not be included in the models. Additionally, the daily occupancy of the close-up pen was recorded for everyday of the study. From the daily THI and close-up pen occupancy values, the THI21 and the average occupancy of the close-up pen for the 21 days before the calving date (OCC21) were calculated for each cow.

### Milk performance and udder health data

Data regarding lactation number, calf sex, twin pregnancy, age at calving (only for primiparous cows), average daily milk yield in the 4 days before dry-off, dry period length, previous calving interval, average daily milk yield during the previous calving interval, and whether colostrum was collected in the morning or afternoon milking were retrieved from the herd management software (DairyPlan C21, GEA Farm Technologies, Düsseldorf, Germany).

Udder health data (number of monthly DHI samplings with SCC linear score > 4 during the previous lactation and the SCC linear score from the last monthly DHI sampling in the previous lactation) was retrieved from the DHI testing monthly reports. Regarding SCC (cells/mL × 1,000), these were analyzed by flow cytometry (Fossomatic, Foss).

### Colostrum collection

Cows on this farm were milked three times daily, but first-milking colostrum collection occurred only at the end of the morning (10:30 a.m.) and afternoon (06:00 p.m.) milkings. Cows calved throughout the night before and early morning had their colostrum collected at the end of the morning milking, whereas cows calving throughout the day had their colostrum collected at the end of the afternoon milking.

First-milking colostrum was collected into a 30-liter milk bucket with a scale (151052- GEA Farm Technologies, Düsseldorf, Germany) in the parlor following milking preparation identical to routine parlor practices but with manual cluster removal, according to farm practices. Colostrum yield was weighed, and total solids were estimated with a colostrum refractometer (KRUUSE, Langeskov, Denmark) and recorded as Brix scale (%Brix). Colostrum yield and total solids were then recorded for each cow.

### Data curation

Environmental and cow-level data were compiled in Microsoft Excel. Lactations were included if complete data were available for colostrum yield, colostrum total solids (%Brix), relevant environmental (THI21, OCC21) and cow-level variables. These cow-level variables included the average daily milk yield in the 4 days before dry-off, dry period length, and previous calving interval, which were calculated from recorded dates (dry-off and consecutive calving dates, respectively). The average daily milk yield during the previous interval was calculated by dividing the total milk yield by the interval length. Records were excluded if the cow died before calving or before colostrum collection. Colostrum total solids were measured using a %Brix refractometer, a common but indirect method for estimating immunoglobulin concentration that may introduce some measurement bias. Because the exact time from calving to colostrum collection was not recorded, the milking session (morning or afternoon) was used as a proxy, which may have introduced additional variability.

### Statistical analyses

Data analysis was performed using SAS (SAS 9.4, SAS Institute Inc., Cary, NC). Associations between environmental, milk performance, and udder health variables and the yield and total solids content of first-milking colostrum were analyzed using general linear models (PROC GLM) for primiparous cows, and mixed models (PROC MIXED) for multiparous cows, with ‘cow’ included as a random effect to account for repeated lactations over time. For multiparous cows, the models also included milk performance and udder health records from the previous lactation, which were not available for primiparous cows.

For lactations of primiparous cows (*n* = 583), the independent variables included in the models were THI21, OCC21, calf sex, age at first calving, and whether colostrum was collected in the morning or afternoon milking. Calf sex (M/F) and whether colostrum was collected in the morning or afternoon milking (M/A) were included in the models as categorical variables and THI21, OCC21, and age at first calving were included as continuous variables.

For lactations of multiparous cows (*n* = 1148), the independent variables included in the models were THI21, OCC21, calf sex, twin pregnancy, lactation number, average daily milk yield in the 4 days before dry-off (MYDRY), dry period length, previous calving interval, average daily milk yield during the previous calving interval, number of DHI > 4LS, LLS, and whether colostrum was collected in the morning or afternoon milking. Calf sex, twin pregnancy (Y/N), lactation number (2, 3, 4, 5, 6+), and whether colostrum was collected in the morning or afternoon milking were included in the models as categorical variables. THI21, OCC21, MYDRY, dry period length, previous calving interval, average daily milk yield during the previous calving interval, DHI > 4LS, and LLS were included as continuous variables. Although potentially useful, interaction terms were not included to maintain model simplicity and improve the interpretability of the main effects.

As suggested by [46], manual backward elimination with a P-value criterion of 0.157 without a preceding univariable prefiltering was performed to reach the final models. The assumption of homoscedasticity was evaluated visually using the plots of studentised residuals against predicted values, and the assumption of normality of residuals was evaluated using the Q-Q plots. All results are presented as least square means ± standard error of the mean (SEM). In the final models, differences were considered significant when *P* < 0.05, whereas a tendency was defined as 0.05 < *P* < 0.10.

The final model assessing colostrum yield in primiparous cows was specified as follows:

Y*ij* = µ + M*i* + ε*ij*

Where Y*ij* is the colostrum yield (kg) of the cow *j* collected in the Milking *i*, µ is the overall mean, M*i* is the fixed effect of Milking collection, and ε*ij* is the residual error.

The final model assessing colostrum yield in multiparous cows was specified as follows:

*Y*_*ijklmno*_ = *µ* + M_*i*_ + *β*_1_THI_*j*_ + *β*_2_DPL_*k*_ + *β*_3_DPL^2^_*k*_ + *β*_4_MYDRY_*l*_ + *β*_5_PCI_*m*_ + LN_*n*_ + *c*_*o*_ + *ε*_*ijklmno*_

Where *Y*_*ijklmno*_ is the colostrum yield (kg) for the observation corresponding to the levels of all factors for cow *o*, *µ* is the overall mean, M_*i*_ is the fixed effect of Milking collection category *i*, *β*_1_ is the regression coefficient of THI21, *β*_2_ is the regression coefficient of the linear term of DPL, *β*_3_ is the regression coefficient of the quadratic term of DPL, *β*_4_ is the regression coefficient of MYDRY, *β*_5_ is the regression coefficient of PCI, LN_*n*_ is the fixed effect of LN category *n*, *c*_*o*_ is the random effect of cow *o*, and *ε*_*ijklmno*_ is the residual error.

The final model assessing colostrum solid content in primiparous cows was specified as follows:

Y*i* = µ + βO*i* + ε*i*

Where Y*i* is the colostrum solid content (%Brix) of the cow *i*, µ is the overall mean, β is the regression coefficient for OCC21, O*i* is the occupancy level for the cow *i*, and ε*i* is the residual error.

The final model assessing colostrum solid content in multiparous cows was specified as follows:

*Y*_*ijkl*_ = *µ* + M_*i*_ + *β*_1_O_*j*_ + *β*_2_DPL_*k*_ + LN_*l*_ + *c*_*m*_ + *ε*_*ijkl*_

Where *Y*_*ijkl*_ is the colostrum solid content (%Brix) for the observation corresponding to the levels of all factors for cow *m*, *µ* is the overall mean, M_*i*_ is the fixed effect of Milking collection category *i*, *β*_1_ is the regression coefficient of OCC21, *β*_2_ is the regression coefficient of the linear term of DPL, LN_*l*_ is the fixed effect of LN category *l*, *c*_*m*_ is the random effect of cow *m*, and *ε*_*ijkl*_ is the residual error.

## Data Availability

The datasets collected/generated during and/or analyzed during the current study are available on reasonable request to the corresponding author.

## Abbreviations

DHI: Dairy Herd Information
DHI > 4LS: Number of monthly Dairy Herd Information tests with somatic cell count (SCC) linear score (LS) ≥ 4 during the previous lactation
Ig: Immunoglobulins
LLS: Linear Score of Somatic cell count in the last Dairy Herd Information test before dry-off
LS: Linear Score
MYDRY: Average daily milk yield in the 4 days before dry-off
OCC21: Average occupancy of the close-up pen for the 21 days before the calving date
SCC: Somatic cell count
THI: Temperature-humidity index
THI21: Average daily maximum THI for the 21 days before the calving date
TPI: Transfer of passive immunity
